# A gastrointestinal stromal tumour presenting incidentally with haemorrhage and perforation associated with a Meckel's diverticulum: a case report

**DOI:** 10.4076/1752-1947-3-7423

**Published:** 2009-07-23

**Authors:** Richard Woolf, Natalie Blencowe, Karim Muhammad, David Paterson, Geoff Pye

**Affiliations:** 1 Department of Coloproctology, Bristol Royal Infirmary, Bristol, UK; 2 Department of Histopathology, Weston General Hospital, Weston-super-Mare, UK; 3 Department of Surgery, Weston General Hospital, Weston-super-Mare, UK

## Abstract

**Introduction:**

This is the first reported case of perforation and haemorrhage of a Meckel's diverticulum leading to the incidental finding of a gastrointestinal stromal tumour within the diverticulum. Meckel's diverticulum is the most common congenital abnormality of the gastrointestinal tract, however, when symptomatic, it is often misdiagnosed at presentation. Common complications presenting in adults include bleeding, obstruction, diverticulitis and perforation. Tumours within a Meckel's diverticulum are a rare but recognised complication. We discuss the management of a gastrointestinal tumour within the diverticulum.

**Case presentation:**

A 59-year-old Caucasian man presented with acute right iliac fossa pain with localized peritonism. At surgery, he was found to have a perforated and haemorrhagic Meckel's diverticulum, associated with a gastrointestinal stromal tumour within the apex of the diverticulum. The absence of necrosis and a low mitotic rate indicated primary resection with subsequent computed tomography surveillance to be the most appropriate management strategy.

**Conclusion:**

We report a unique triad of complications associated with the presentation of a Meckel's diverticulum. This article reviews this common congenital abnormality and discusses the management of a gastrointestinal tumour. Meckel's diverticulum will mimic other intra-abdominal pathologies in presentation and should therefore often be considered as a differential diagnosis.

## Introduction

This is the first reported case of perforation and haemorrhage of a Meckel's diverticulum leading to the incidental finding of a gastrointestinal stromal tumour within the diverticulum. Meckel's diverticulum is the most common congenital abnormality of the gastrointestinal tract, however, when symptomatic, it is often misdiagnosed at presentation. Common complications presenting in adults include bleeding, obstruction, diverticulitis and perforation. Tumours within a Meckel's diverticulum are a rare but recognised complication.

## Case presentation

A 59-year-old Caucasian man presented with peri-umbilical pain that had localized to the right iliac fossa. On examination, he was tender in the right iliac fossa, with localized peritonism. His white cell count was 10.2 × 10^9^ (neutrophils 8.1 × 10^9^) and with C-reactive protein (CRP) <5. Acute appendicitis was diagnosed clinically and a diagnostic laparoscopy performed.

**Figure 1:**
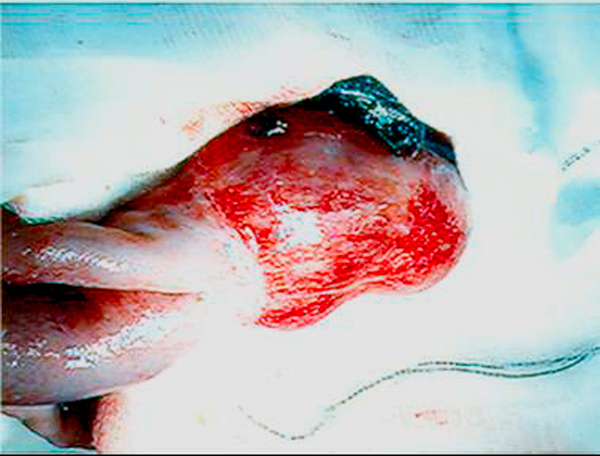
**Intra-operative photograph demonstrating Meckel's diverticulum and overlying thrombus**.

**Figure 2:**
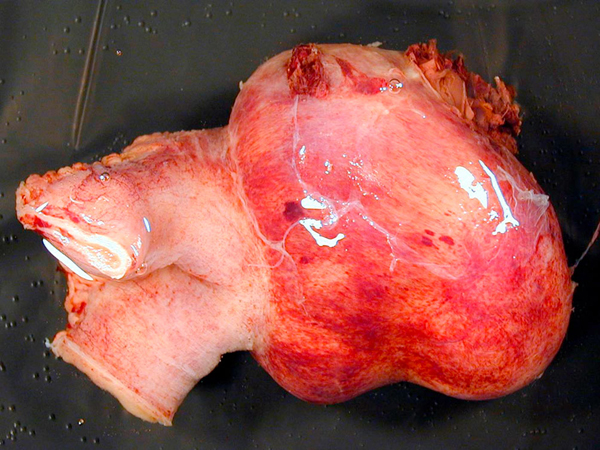
**Resected specimen**.

**Figure 3:**
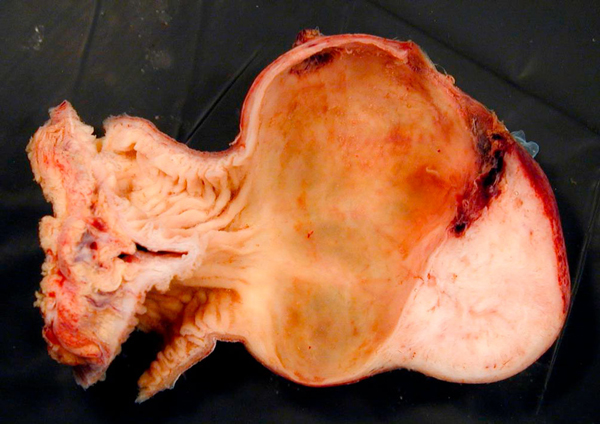
**Resected specimen in cross-section demonstrating the perforated wall of the Meckel's diverticulum at the superior margin and the gastrointestinal stromal tumour at the apex of the diverticulum**.

**Figure 4:**
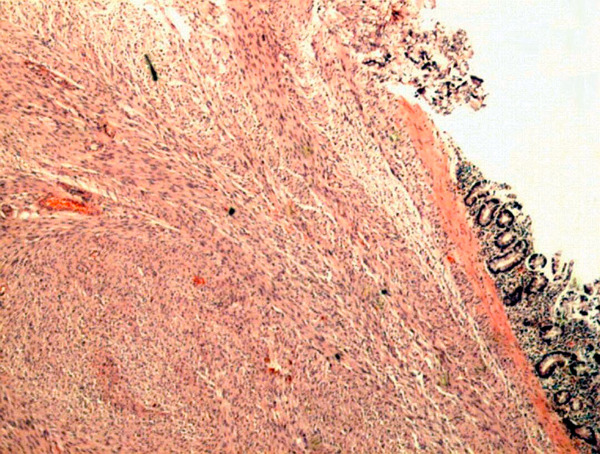
**Light micrograph of histological specimen demonstrating full thickness tumour of the bowel wall extending to the serosal surface (haematoxylin and eosin stain; ×100)**.

A perforated Meckel's diverticulum was found, associated with free intra-abdominal fluid and haemorrhage. At subsequent laparotomy, 75 mm of small bowel was resected and primary anastamosis was performed. Histology confirmed a Meckel's diverticulum and with a 25 mm area of perforation (Figures 1-3). An incidental finding was a 45 mm nodule at the apex of the diverticulum with the following features:

1.Â Full thickness tumour of the bowel wall, extending to serosal surfaces (Figure 4).

2.Â No areas of tumour necrosis.

3.Â Less than one mitotic figure in 10 × 40 high powered fields.

4.Â Interlacing bundles of spindle cells with elongated blunt ended nuclei.

5.Â Some nuclear variability and tumour giant cells present.

**Figure 5:**
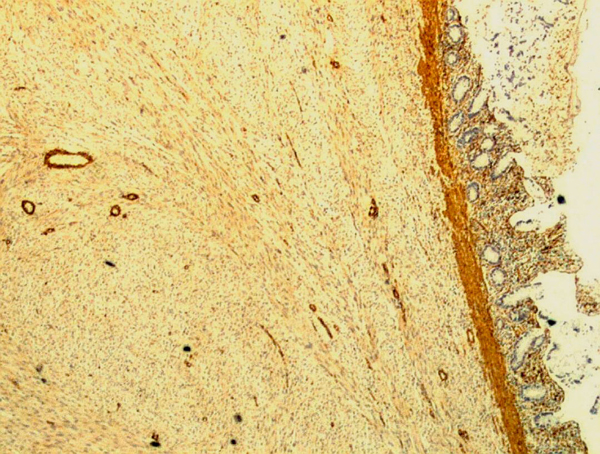
**Light micrograph with smooth muscle actin staining showing uptake by tumour tissue (×100)**.

**Figure 6:**
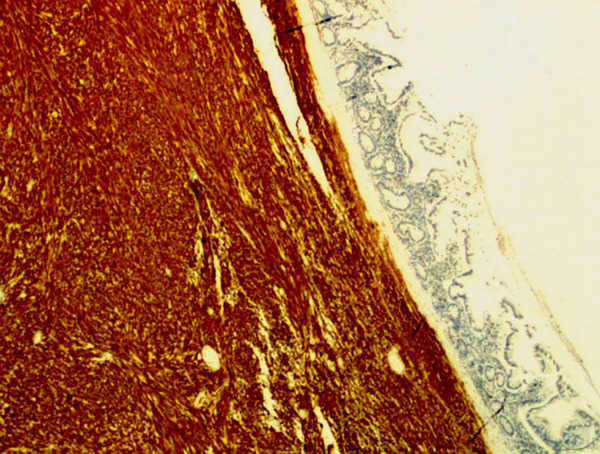
**Light micrograph with CD117 staining showing uptake by tumour tissue (×100)**.

6.Â Positive for CD117 and smooth muscle actin (Figures 5 and 6), negative for S100 protein and cytokeratin.

This was, therefore, confirmed to be a GIST. Resection margins were found to be complete. The patient received 72 hours of intravenous antibiotics and made a good recovery. Surveillance abdominal computed tomography (CT) scan at one year was unremarkable.

## Discussion

Meckel's diverticulum is a congenital abnormality that arises at the site of the vitelline duct, which in the embryo connects the primitive gut to the yolk sac. If this fails to obliterate by the seventh week of gestation, congenital defects can persist that include umbilical sinus, omphalomesenteric fistula, enterocyst, fibrous band and most commonly Meckel's diverticulum. The diverticulum is a true diverticulum containing all layers of the intestinal wall and most commonly arises from the antimesenteric aspect of the ileum, proximal to the ileocaecal valve. It has an independent blood supply from a remnant of the vitelline artery, a branch of the superior mesenteric artery.

The diverticulum is commonly described by the rule of '2s': occurring in 2% of the population, approximately 2 inches long, arising within 2 feet of the ileocaecal valve, commonly affecting children less than 2 years of age and occurring twice as often in men [1]. Although approximate guidelines describe this anatomical variant, they are not accurate. The diagnosis of a Meckel's diverticulum is often incidental at laparotomy or laparoscopy and its prevalence is only approximated at 2%. Some autopsy studies suggest that the true percentage may be higher at 4.5% [1]. Ninety percent of the diverticula are less than 10 cm long but cases have been reported of up to 100 cm [2]. Meckel's diverticula occur in the terminal ileum but their distance from the ileocaecal valve has been found to increase with age with an average distance of 34 cm in children under the age of 2 years, but 64 cm in adults [3].

Meckel's diverticulum can either be an incidental finding or present symptomatically. It is thought that between 4.2% and 6.4% become symptomatic, with the incidence falling with increasing age [4]-[6]. The mean age of presentation of symptomatic Meckel's diverticulum is 31 years with a male to female ratio of 3:1 (in both adult and paediatric groups), however, the incidental diagnosis has a more equal sex distribution in adults [6]. In a large series of 1476 cases at the Mayo Clinic, Park *et al*. report the most common presentations of symptomatic Meckel's diverticula in adults to be bleeding (38%), obstruction (34%), diverticulitis (28%) and perforation (10%) [6]. Perforation is most common secondary to ulceration of ectopic gastric mucosa, foreign bodies or Littre's hernia [7]. Due to the rarity of this anatomical abnormality, symptomatic Meckel's diverticula are misdiagnosed in approximately 90% of cases and acute appendicitis is the usual pre-operative diagnosis [8].

Tumours are reported to occur in 0.5% and 3.2% of symptomatic Meckel's diverticula [8]. In a review of reported cases since 1965, Hager *et al*. state the prevalences of tumours of Meckel's diverticula to be: carcinoid tumour (31.5%), leiomyosarcoma (25.5%), adenocarcinoma (11.4%) and leiomyoma (9.4%) with overall malignancy in 77% of cases [7]. The definition of a gastrointestinal stromal tumour (GIST) has varied since the first use of the term in 1983. Originally, it encompassed gastrointestinal non-epithelial neoplasms lacking the immunohistochemical features of Schwann cells and did not have the ultrastructural characteristics of smooth muscle cells [9]. Using this original classification of GIST, 42% of all tumours and 41% of malignant tumours of Meckel's diverticula would be classified as GIST [7]. However, GIST is now recognised as a separate tumour entity and is defined as a spindle cell, epithelioid or pleiomorphic mesenchymal tumour of the gastrointestinal tract that strongly expresses the KIT (CD 117) protein and may harbour mutations of the type III tyrosine kinase receptor gene (either *KIT* or *PDGFRA*) [10].

GIST accounts for 0.1% and 3% of all gastrointestinal neoplasms, most commonly occurring in the stomach or small bowel, and is now the most common sarcoma of the small intestine [10]. Small bowel GISTs have a range of presenting features, including abdominal pain, an abdominal mass, gastrointestinal bleeding, small bowel obstruction, weight loss, fever, abscess or perforation [7].

There are little prognostic data regarding GISTs and current prognostic indicators are based on consensus guidelines. The most important adverse factors are thought to be a tumour diameter of greater than 5 cm and a high mitotic count exceeding five mitotic figures per 50 high powered fields on light microscopy [10,11]. Other suggested factors indicative of poor prognosis include tumour perforation, tumour necrosis, high cellularity and marked pleiomorphism [10]. The case reported by us has a low risk of recurrence based on a maximum diameter of 4.5 cm, a low mitotic count of less than one mitotic figure in 10 × 40 high powered fields, and no evidence of necrosis. Importantly, the perforation of the diverticulum was also not associated with the tumour nodule. Surgery is considered the standard treatment for non-metastatic GIST with en-bloc resection and clear margins. Study data on GISTs presenting in the United States between 1992 and 2000 state a 5-year survival of 50-60% after complete resection of the localized primary tumour [12]. There is little evidence supporting local/regional lymphadenectomy as GISTs rarely metastasize to lymph nodes [10]. Targeted therapy with Imantinib, a KIT tyrosine kinase inhibitor, is considered the standard treatment for metastatic GIST [10].

## Conclusion

Since 1978, there have been approximately 10 reported cases of tumour-associated perforation of a Meckel's diverticulum, however, only two of these were histologically classified as GISTs [7,13]. Our case is the first reported patient with perforation of a Meckel's diverticulum with frank intra-abdominal haemorrhage that led to the incidental discovery of the separate pathology of GIST within the diverticulum. Meckel's diverticulum can mimic other intra-abdominal pathologies in presentation and should therefore be considered as a differential diagnosis.

## Abbreviations

CRP: C-reactive protein; CT: computed tomography; GIST: gastrointestinal stromal tumour.

## Consent

Written informed consent was obtained from the patient for publication of this case report and any accompanying images. A copy of the written consent is available for review by the Editor-in-Chief of this journal.

## Competing interests

The authors declare that they have no competing interests.

## Authors' contributions

RW conceived the original article design, drafted the original manuscript reviewing current literature and corresponded with the editorial team. NB summarized the case and helped substantially with the drafting and critiquing of the original manuscript. KB and GP were the surgeons who operated on the patient, and helped substantially with critiquing of the original manuscript. DP examined the histological specimens and provided the histological diagnosis. All authors read and approved the final manuscript.
